# Cancer cell reprogramming: a promising therapy converting malignancy to benignity

**DOI:** 10.1186/s40880-019-0393-5

**Published:** 2019-08-29

**Authors:** Lanqi Gong, Qian Yan, Yu Zhang, Xiaona Fang, Beilei Liu, Xinyuan Guan

**Affiliations:** 1 Department of Clinical Oncology, The University of Hong Kong, Hong Kong, 999077 P.R. China; 2 State Key Laboratory for Liver Research, The University of Hong Kong, Hong Kong, 999077 P.R. China

**Keywords:** Cancer cell reprogramming, Transcription factor, Small molecule, MicroRNA, Exosome, Malignancy, Benign, Pluripotency, Cancer stem cell, Induced pluripotent stem cell

## Abstract

In the past decade, remarkable progress has been made in reprogramming terminally differentiated somatic cells and cancer cells into induced pluripotent cells and cancer cells with benign phenotypes. Recent studies have explored various approaches to induce reprogramming from one cell type to another, including lineage-specific transcription factors-, combinatorial small molecules-, microRNAs- and embryonic microenvironment-derived exosome-mediated reprogramming. These reprogramming approaches have been proven to be technically feasible and versatile to enable re-activation of sequestered epigenetic regions, thus driving fate decisions of differentiated cells. One of the significant utilities of cancer cell reprogramming is the therapeutic potential of retrieving normal cell functions from various malignancies. However, there are several major obstacles to overcome in cancer cell reprogramming before clinical translation, including characterization of reprogramming mechanisms, improvement of reprogramming efficiency and safety, and development of delivery methods. Recently, several insights in reprogramming mechanism have been proposed, and determining progress has been achieved to promote reprogramming efficiency and feasibility, allowing it to emerge as a promising therapy against cancer in the near future. This review aims to discuss recent applications in cancer cell reprogramming, with a focus on the clinical significance and limitations of different reprogramming approaches, while summarizing vital roles played by transcription factors, small molecules, microRNAs and exosomes during the reprogramming process.

## Background

**Fig. 1:**
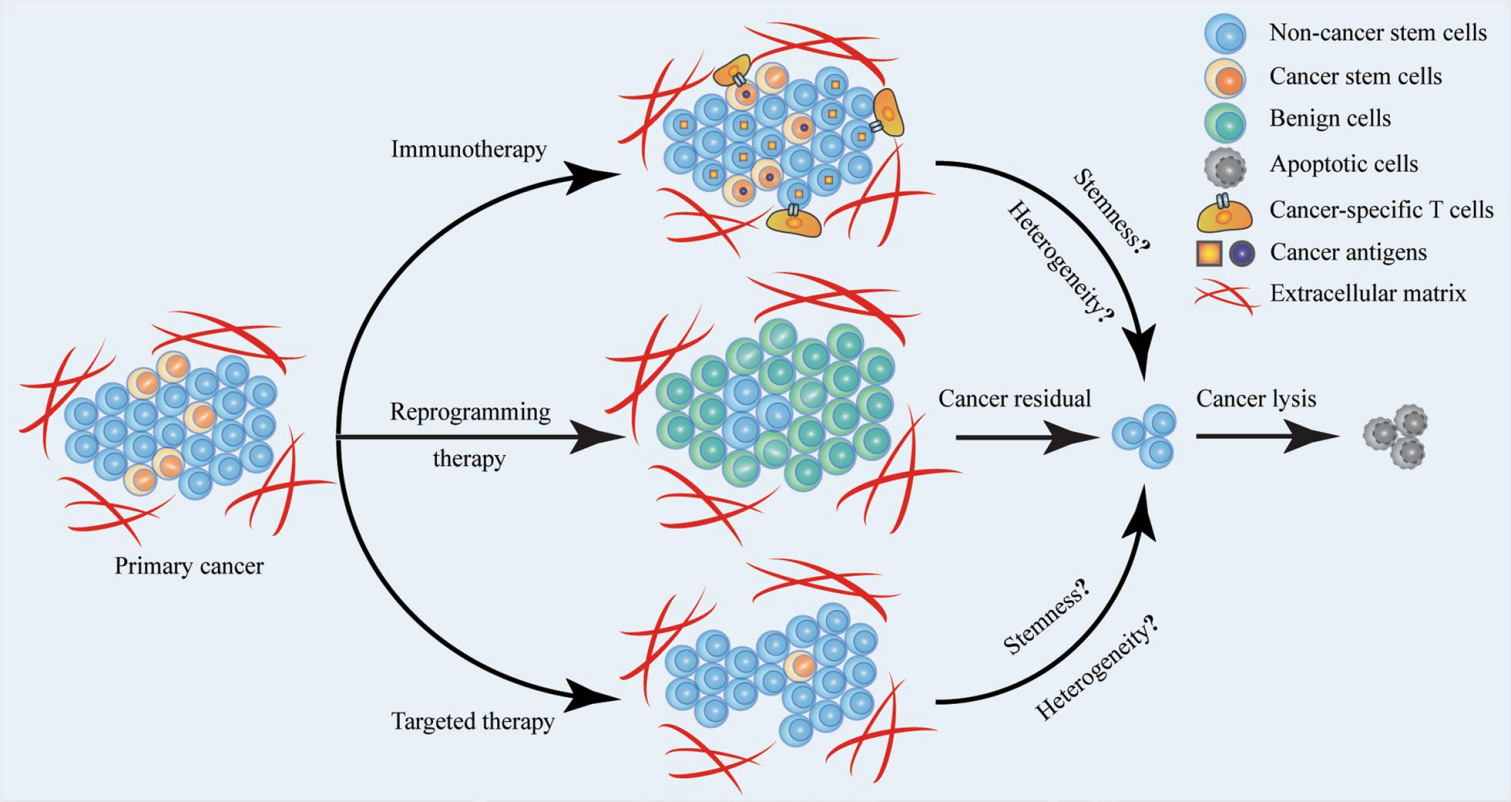
Emerging therapeutic strategies against primary cancer. Researchers and clinicians have explored three mainstay strategies for cancer treatment: regulating the immune responses to cancer cells; reprogramming cancer cells into benign cells; directly eradicating cancer stem cells. Immunotherapy and targeted therapy have better therapeutic performance comparing to conventional chemo-/radio-therapy, but their effects are still suffering from the existence of cancer stem cells and heterogeneity. Cancer cell reprogramming therapy elicits a potential to convert cancer cells into benign cells regardless of cell subtypes. Although cancer cell reprogramming therapy has not entered clinical trials to date, progress still continues

Cancer is responsible for an estimated 9.6 million deaths in 2018 [1, 2]. To date, surgery remains as one of the primary and most effective strategies for early-stage cancers [3, 4]. Whereas, the feasibility and outcomes of surgery highly depend on patient-specific circumstances, including cancer stages and physiological status [5]. More than 50% of patients in stage III and IV will receive conventional chemo- and radio-therapy. However, most of them quickly develop acquired resistance [3, 6]. Although immunotherapy and targeted therapy have emerged as effective strategies in the past few years, their effects have been partially impeded due to cancer heterogeneity and the existence of cancer stem cells (CSCs) [7–9]. Therefore, finding potential treatments that can globally manage cancer remains a crucial task so far (Fig. 1).

**Fig. 2:**
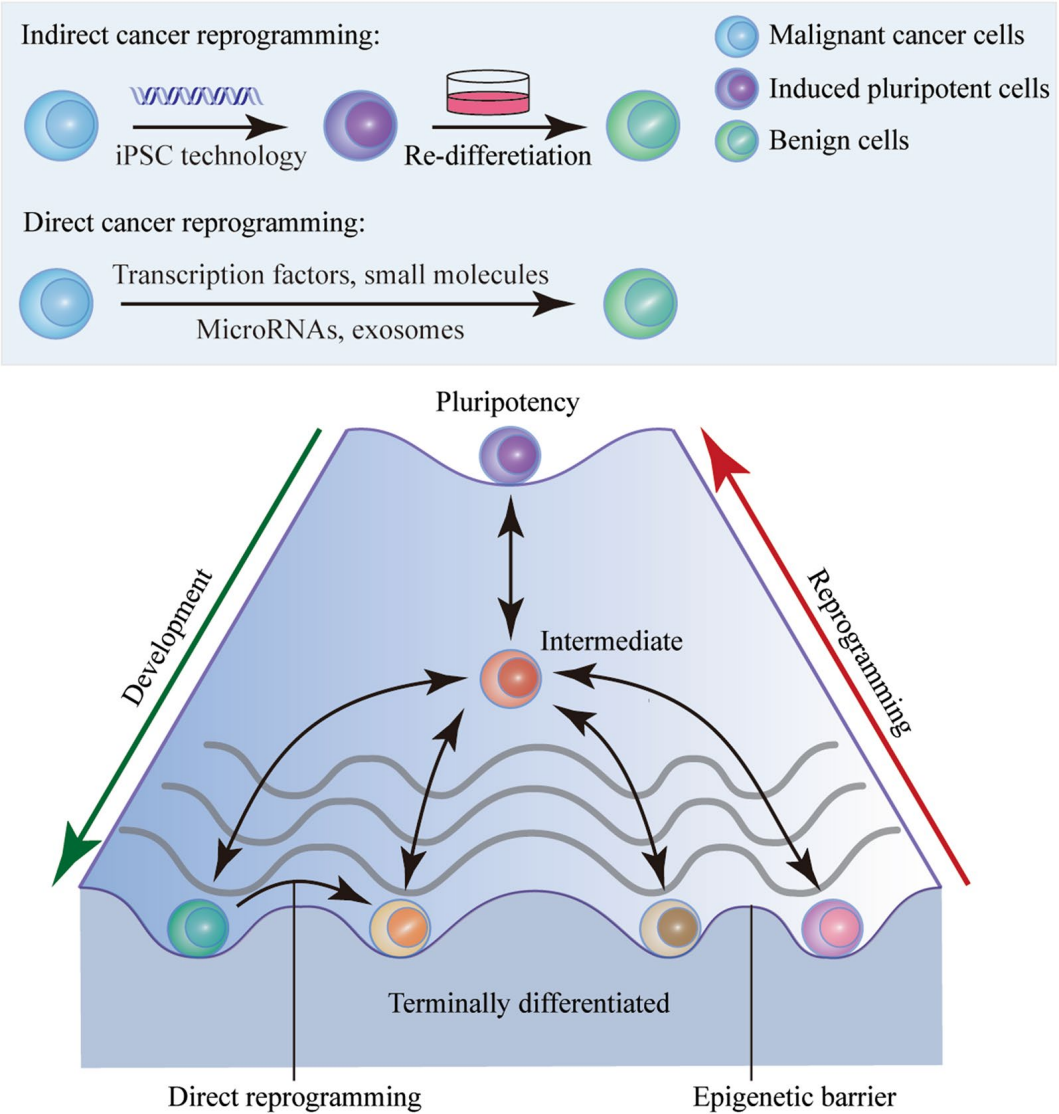
Epigenetic landscape of cell reprogramming and development. Cells undergo extensive epigenetic modifications from pluripotency to a terminally differentiated state. Cell fates have been identified as flexible and reversible, suggesting that terminally differentiated cells, such as cancer cells, are feasible to be reprogrammed back into a pluripotent stage via re-activation of epigenetic barriers. The induced pluripotent stem cells can further differentiate into benign cells with distinct lineages. Unlike indirect cancer cell reprogramming, direct cancer cell reprogramming allows cells to bypass the pluripotent stage so that they can be directly converted into other types of cells by transcription factors, small molecules, microRNAs or exosome

The concept of cellular plasticity was first proposed by Gurdon et al. [10]. They confirmed that terminally differentiated somatic cells could be reprogrammed into other lineages. Cancer cells are also genetically and epigenetically plastic, suggesting that they have the potential to retrieve benign cell functions via re-expression of lineage-specific genes [11]. Therefore, cancer cell reprogramming has emerged as a promising strategy which can induce the transition from malignancy to benignity. It can be achieved through various approaches, including combinatorial delivery of transcription factors, small molecules, microRNAs, and exosomes [12]. During cell reprogramming, DNA methylation and histone modifications, cell behaviors, and gene expression profiles can undergo dramatic alterations [13–16] (Fig. 2). Much effort has been focused on optimizing reprogramming protocols and deciphering molecular mechanisms to achieve high efficiency, safety, and specificity [17]. The rapid evolution of cancer cell reprogramming has provided substantial insights into biomedical science and translational medicine [18]. Here, we first review the varied approaches that induce cancer cell reprogramming into CSCs and second, concentrate on the recent applications of facilitating reprogramming therapy for in vitro*/*in vivo cancer transition to benignity.

## Reprogramming cancer cells into CSCs

Re-activation of the epigenetically silenced regions is one of the frequently used approaches to induce cancer cell reprogramming [13]. DNA methylation, chromosome remodeling, histone methylation, and acetylation are major epigenetic modifications that determine cellular plasticity [13, 19]. Plasticity-associated genes in terminally differentiated cells usually embed in silenced chromatin blocked by nucleosomes [20]. The silenced regions can be re-activated by a subset of transcription factor-encoding genes via regulation of the transcription network [21]. Transcription factors that are highly expressed in germ cells and embryonic stem cells (ESCs) have been considered responsible for such manipulation of cellular plasticity [22, 23]. Therefore, extensive analysis of mouse ESCs has been performed to identify substantial transcription factors that are strongly associated with cancer stemness and infinite proliferation [24–26].

In the year 2006 and 2007, Yamanaka et al. [27, 28] proved that mouse and human fibroblasts could be reprogrammed into induced pluripotent stem cells (iPSCs) by virus-mediated transduction of Kruppel-like factor 4 (KLF4), Octamer-binding transcription factor 3/4 (Oct-3/4), Sex-determining region Y-box 2 (SOX2) and c-Myc, later referred to as Yamanaka factors. The invention of transcription factor-mediated iPSC technology has led to substantial breakthroughs in the research of CSCs [29–32]. The concept of CSCs was raised from clinical and experimental observations in which it was found that a small subpopulation of cancer cells possesses pluripotent characteristics including self-renewal and differentiation potential. They could lead to cancer development, relapse, and drug resistance [33]. However, CSCs normally constitute of 0.05–1% of the cancer population, and they are difficult to be isolated and characterized [34]. Therefore, the molecular mechanisms of how CSCs cause varied malignancies remains poorly understood [35]. It has reported that reprogrammed CSCs exhibit similar capability to initiate tumor growth, metastasis, and chemo-/radio-resistance and possess similar gene profiles with primary CSCs [36]. Hence, cancer cell reprogramming can serve as a useful platform to comprehensively study CSC-associated mechanisms, including the origin and molecular functions of CSCs [12].

Through the Yamanaka factor-mediated reprogramming, varied types of cancer cells including, leukemia, breast, bladder, liver, prostate, and pancreatic cancer cells, were stably reprogrammed into CSCs with enhanced expressions of stemness-related genes including SOX2, Nanog homeobox (NANOG), stage-specific embryonic antigen-1 (SSEA-1), T cell receptor alpha-1–60 (TRA-1–60), and T cell receptor alpha-1–81 (TRA-1–81) [37–41]. Nevertheless, the efficiency of reprogramming from cancer cells into CSCs remained relatively low due to the existence of genetic and epigenetic barriers [37, 38]. This phenomenon has also been observed in somatic cell reprogramming [27, 28]. Yamanaka et al. [27, 28] found that only 0.02% of fibroblasts became iPSCs, and later they discovered that the success rate of reprogramming was primarily limited by introduction efficiency and genetic signatures of the targeted cells. In addition to the low in vitro reprogramming efficiency, Yamanaka factors have also been reported to have oncogenic potentials in varied cell types [42–45]. Therefore, in vivo introduction of Yamanaka factors might result in cancer progression and such safety concern has been raised against future clinical applications.

To improve the efficiency and safety of cancer and somatic cell reprogramming, many efforts have been put into finding potential small biochemical molecules that can enhance reprogramming efficiency or replace some of the vital transcription factors [46]. During the last decade, various small molecules including histone deacetylases, methylases, and demethylases inhibitors, DNA methyltransferase inhibitors, and Wnt and Rho-associated protein kinase (ROCK) pathway regulators, have been proven to be effective in inducing reprogramming of terminally differentiated and cancer cells [47–50]. For instance, valproic acid (VPA), a histone deacetylase inhibitor, increased the efficiency of transcription factor-mediated cell reprogramming from 0.50% ± 0.06% to 11.8 ± 2.2% Oct4-GFP^+^ iPSC colonies (> 100-fold change), indicating chromatin modification is one of the major rate-determining steps during reprogramming [51]. Several small molecules have also been identified as being responsible for improving in vitro reprogramming efficiency, including 2-[3-(6-methyl-2-pyridinyl)-1*H*-pyrazol-4-yl]-1,5-naphthyridine (RepSOX2, E-616452), and Oct4-activating compound 1 (OAC1), which facilitate the mesenchymal-epithelial transition (MET) and activate the stemness-associated promoter regions of mature fibroblasts [52, 53]. Use of small molecules still relies on introducing transcription factors into cells, so that it remains challenging to break through the efficiency threshold due to insufficient gene delivery and limitations in cellular uptake [54]. More details about the introduction of transcription factors with combinatorial small molecules in cancer cell reprogramming to CSCs were previously reviewed [55, 56].

## Reprogramming cancer cells to benign cells

### Transcription factor-mediated cancer cell reprogramming: a pioneer

Since treatment against cancer recurrence, metastasis, and resistance remain challenging in clinics, the implementation of cancer gene therapy has remained a thriving and demanding option that might overcome such difficulties [57]. It is well recognized that benign cells can become cancer cells after a malignant transition, but whether cancer cells can be genetically and epigenetically reversed back to a benign phenotype remains unclear [58]. Transcription factor-mediated reprogramming has recently emerged as an in vitro approach to enable cancer cells to retrieve benign functions.

As previously discussed, iPSC technology, as a sophisticated reprogramming approach, has not been only exploited to induce a somatic transition from terminally differentiated somatic cells to pluripotency but also been used to generate CSCs for oncogenic characterization [21, 38]. In addition, iPSC technology has also been frequently used to induce reprogramming from cancer cells to pluripotent cells with a benign phenotype. In 2009, Utikal et al. [59] reprogrammed human melanocytes and mouse melanoma cell line to iPSCs with a benign phenotype by the introduction of Yamanaka factors with efficiency ranging from 0.05% to 0.1%. R545 melanoma cell line-derived iPSCs exhibited endogenous expression of Oct4, Klf4 and c-Myc, demethylation of the Oct-4 and NANOG promoters and the loss of in vivo tumorigenicity. Upon discontinuation of doxycycline-inducible lentiviral expression of Yamanaka factors by withdrawing doxycycline, mouse chimeras derived from the reprogrammed melanoma cells had maintained benignity and did not form visible tumor at 5 months of age, indicating the reprogrammed cells underwent normal differentiation process to produce benign cells in vivo.

In 2010, Miyoshi et al. [60] found that the expression of pluripotency-associated genes, such as NANOG, stage-specific embryonic antigen-4 (SSEA-4), TRA-1–60, and TRA-1–81 was elevated after introducing Yamanaka factors into pancreatic, liver and colorectal cancer cells. Reprogramming reversed DNA and histone methylation in specific promoter regions to re-express pluripotency-associated genes so that the reprogrammed cancer cells were able to develop patterns similar to ectoderm, mesoderm, and endoderm. Besides, the pluripotent cancer cells possessed higher sensitivity to chemotherapeutic agent 5-fluorouracil (5-Fu), leading to a potential clinical significance to revoke acquired chemo-/radio-resistance via cancer cell reprogramming. In addition, the reprogrammed cancer cells were able to differentiate into various lineages, including epithelial, mesenchymal, and neuronal cells (collectively referred to as post pluripotent cancer cells). The post pluripotent cancer cells were less malignant compared to parental cancer cells in vitro and were free of tumorigenic potential based on tumor formation assay in NOD/SCID mice. However, the reprogramming efficiency from cancer cells to pluripotent cancer cells remains low, suggesting that there only a minority of tumor cells could be successfully reprogrammed into pluripotent cancer cells. Later studies also demonstrated that other combinatorial transcription factors, such as Lin-28 homolog (LIN28), Oct-4, SOX2, and NANOG, were also able to reprogram lung adenocarcinoma and gastrointestinal cancer into iPSCs with alleviated tumorigenicity and metastatic potential [61–63]. Transcription factor-mediated reprogramming can be further directed, by a variety of differentiation-associated factors, to form functional cells of diverse lineages [64]. Although such reprogramming approach is feasible and ethically-acceptable to re-activate the post-epigenetic state of cancer cells back into a benign pluripotent state [65], the efficiency and safety of cancer cell reprogramming mediated by transcription factors remain a challenging task to be solved before it becomes a promising therapy for cancer [29].

Combinatorial pluripotency-associated transcription factors have shown proven capabilities to reprogram cancer cells to uiPSCs with the potential to further differentiate into normal cells. In addition, investigators have recently found that lineage-specific factors can directly reprogram cancer cells into functional somatic cells by bypassing the pluripotent stage, which could decrease the risk for malignant transformation of induced pluripotent cancer cells [66].

Breakpoint cluster region (BCR)-Abelson murine leukemia viral oncogene homolog 1 (ABL1)^+^ precursor B-cell acute lymphoblastic leukemia (B-ALL) is characterized by blockade of B-cell differentiation. Hence, reprogramming of BCR-ABL1^+^ B-ALL into the non-leukemic cells has been considered as an excellent strategy to overcome the differentiation blockade [67, 68]. A previous study has shown that CCAAT/enhancer-binding protein alpha (C/EBPα), a transcription factor associated with the development of ALL, can induce a cellular transition from murine B lineage cells to macrophages with approximately 100% efficiency [69]. This earlier work has led to the consideration of whether C/EBPα could also be used to reprogram cancer with B cell lineages to functional macrophages. In 2013, Rapino et al. [70] successfully reprogrammed human lymphoma and leukemia B cell lines to macrophage-like cells by introduction of C/EBPα. According to the analysis of more than 20 human lymphoma and leukemia B cell lines, 80% of the cells could be partially or entirely reprogrammed to macrophage-like cells. The reprogrammed lymphoblastic leukemia B cells showed less tumorigenicity in vitro, with the up-regulation of macrophage-associated markers and down-regulation of B cell-associated markers. Experiments in murine models also confirmed that no tumor was formed after the injection of C/EBPα-infected lymphoid leukemia cells into immunodeficient mice. Although the majority of the analyzed lymphoma and leukemia cell lines underwent reprogramming at least partially or transiently, only two cell lines with a higher endogenous expression of C/EBPα effectively sustained the cellular transition to macrophage-like cells, indicating the success rate of cancer cell reprogramming highly depends on the endogenous expression of C/EBPα [71, 72].

Previous studies have focused on introducing a single nuclear transcription factor to alleviate the tumorigenicity of not only B cell-associated malignancies but also hepatocellular carcinoma (HCC) [73–75]. To successfully reprogram cancer cells into cells with normal functions, it is necessary for various nuclear transcription factors to work cooperatively [76]. Whether there is a specific formula of transcription factors that can effectively induce cancer transition from malignancy to benignity with high efficiency and safety remains elusive. Recent advances in single-cell RNA sequencing have enabled investigators to obtain more comprehensive profiling in different cancer cells, and an increasing number of transcription factor candidates have been identified and characterized to improve the efficiency of cancer cell reprogramming [77–79].

In 2014, Huang et al. [80] found that a combination of transcription factors including hepatocyte nuclear factor 1 alpha (HNF1A), hepatocyte nuclear factor 3 alpha (HNF3A) and forkhead box protein A3 (FOXA3) played a significant role in reprogramming human fibroblasts into hepatocyte-like cells. Then in 2019, Cheng et al. [76] demonstrated that the combination of HNF1A, HNF4A and FOXA3 could also induce direct reprogramming of HCC into hepatocyte-like cells with normal functions including albumin secretion, glycogen synthesis, low-density lipoprotein uptake as well as metabolism control and detoxification. In this study, adenovirus was used to synergistically introduce HNF1A, HNF4A, and FOXA3 into HCCLM3 and Huh7 cell lines. Based on its intrinsic hepatotropism, as compared to iPSC reprogramming, adenovirus-mediated infection induced approximately 100% HCC cells to express the selected transcription factors which significantly improved the infection and reprogramming efficiency. The combinatorial transcription factors induced there-expression of hepatocyte-associated genes and morphological changes in both HCCLM3 and Huh7 cell lines, indicating a simultaneous effect of HNF1A, HNF4A, and FOXA3 in HCC reprogramming. Reprogrammed hepatocytes showed gradual gaining of hepatocyte functions and losing of in vitro tumorigenic characteristics. For instance, the reprogrammed hepatocytes from HCCLM3 cell lines exhibited a significant increase of albumin (ALB) expression and decrease of alpha-fetoprotein (AFP) expression. The results from the colony-forming assay, migration assay, and spheroid formation assay also indicated that the proliferation and migration abilities, as well as the number of liver CSCs were decreased. The results of cDNA microarray confirmed that the reprogrammed hepatocyte-like cells were genetically similar to primary human hepatocytes. Murine models also showed that the reprogrammed hepatocyte-like cells substantially lost in vivo tumorigenicity and were capable of reconstructing the liver structure during regeneration. Further, the epithelial cell adhesion molecule (EpCAM)^+^ subpopulation in the reprogrammed hepatocyte-like cells was significantly decreased, suggesting that cancer cell reprogramming via HNF1A, HNF4A and FOXA3 could effectively eliminate CSCs to prevent cancer recurrence, relapse, and resistance in HCC.

Transcription factor-mediated reprogramming is based on genetic and epigenetic modifications via specific gene delivery [21]. Since Yamanaka et al. successfully exploited the transcription factors to reprogram mouse and human fibroblasts into iPSCs, the reprogramming technique has been further deployed in the development of potential cancer treatments [21, 27, 28]. Nevertheless, controversy regarding the transcription factor-mediated cancer cell reprogramming remained [81]. Several studies have shown that reprogramming from cancer cells to pluripotent cells do not always lead to positive effects. For example, owing to the presence of oncogenes such as c-Myc, KLF4, and SOX2, pluripotent cancer cells possess safety concerns in oncogenesis due to aberrant differentiation [62, 63]. Furthermore, transcription factor-mediated cancer cell reprogramming has addition limitations in terms of cost, introduction efficiency, and in vivo delivery, which have hindered its potential in clinical translation [82]. Cancer initiation and progression are primarily related to genetic mutations and complicated epigenetic alternations, including microRNA regulation, DNA methylation, histone modifications, and chromosome remodeling [83]. Transcription factor-mediated cancer cell reprogramming is highly involved in these complex molecular networks and the underlying mechanism remains largely unexplored.

### Small molecule-mediated cancer cell reprogramming: a game-changer

The advent of transcription factor-mediated cancer cell reprogramming has provided groundbreaking outcomes to prove the feasibility of reprogramming cancer fates [13, 27, 28]. Although the transcription-mediated cancer cell reprogramming is widely recognized as a potentially promising strategy against malignancies, safety and efficacy concerns caused by transgenic modifications remain as a non-negligible blockade [84]. The genetic abnormalities, such as activation of oncogenes or silencing of tumor suppressor genes caused by the insertion of exogenous DNA sequences may jeopardize future clinical translation of cancer cell reprogramming therapy. There has been an alternative approach to replace the viral infection with transient gene delivery using specially designed micro-particles [85], but the transcription factor-mediated cancer cell reprogramming remains risky and technically challenging [82, 83]. Therefore, there is an urgent demand for establishing alternative strategies to induce efficient cancer cell reprogramming. Recently, small molecule-mediated cancer cell reprogramming has proven to be capable of reprogramming terminally differentiated cells into a pluripotent state [47, 86, 87]. More significantly, there are also several studies eliciting that using small molecules to induce cancer cell reprogramming from malignancy to benignity can circumvent some of the limitations in transcription factor-mediated cancer cell reprogramming [88, 89].

Small molecule-mediated reprogramming has distinct advantages, including relatively low cost, simple technique, easily-tunable versatility, permeability, and reversibility [17, 90]. Small molecules can also serve as an excellent candidate to efficiently regulate cellular processes via directly targeting signaling pathways such as the Wnt, Hedgehog, and Hippo pathways [91–93]. It is convenient to manufacture small molecules and scale their throughput to induce reprogramming with different lineages [94]. Moreover, such molecules can be utilized as molecular probes to investigate the underlying changes in molecular signaling during cancer cell reprogramming, which might lead to an improvement in reprogramming efficiency and reduction of the off-target effect [94]. For small molecule-mediated cancer cell reprogramming to succeed, it is necessary to identify and develop small biochemical molecules that can assist cancer cells in overcoming the epigenetic barriers and blockades in various cellular signaling pathways [88, 89]. Since using small molecule-mediated cancer cell reprogramming independently to convert malignancy to benignity remains challenging so far, there is a limited number of studies in the extending frontier [88, 89].

By introducing C/EBPα, Rapino et al. [70] successfully reprogrammed human lymphoma and leukemia B cell lines to macrophage-like cells. The finding leads to a theoretical insight on whether small molecules can also exert effects on reprogramming of lymphoblastic leukemia. In year 2015, McClellan et al. [88] found that myeloid differentiation-inducing cytokines, including FMS-like tyrosine kinase ligand (FLT3L), interleukin 7 (IL-7), interleukin 3 (IL-3), granulocyte-macrophage colony-stimulating factor (GM-CSF), macrophage colony-stimulating factor (MCSF) and myeloid transcription factors such as C/EBPα and PU.1 could efficiently reprogram primary human BCR-ABL1^+^ B-ALL cells into macrophage-like cells. After 2-week exposure to myeloid differentiation-inducing cytokines, 53% of the CD19^+^/CD34^+^ leukemic blasts were found to significantly increase the expression of CD14 and decrease the expression of CD19. The CD14^+^/CD19^−^ subpopulation were sorted and purified to yield > 98% macrophage-like cells with stable CD14 expression. The reprogrammed cells possessed macrophage-like morphology, surface immunophenotypes, gene expression profile, generation of oxidative burst, and phagocytic ability. Furthermore, the reprogrammed cells could significantly alleviate leukemogenicity, manifested by the loss of the capacity to form malignant xenografts in animal models. The results might lead to a feasible strategy that exploits cancer cell reprogramming to treat BCR-ABL1^+^ B-ALL in vivo. Nevertheless, the results generated from leukemic reprogramming suggested in vivo reprogramming was at a preliminary stage as underlying genetic aberrations caused by cytokine induction remains unexplored. Moreover, 5 out of 12 clinical cases showed resistance to CD14^+^ reprogramming. Therefore it is unclear how to prospectively choose patients who would benefit from leukemic reprogramming. More research is entailed to overcome these limitations before it can become an efficient therapeutic strategy against B cell-associated malignancies.

In year 2019, Ishay-Ronen et al. [89] successfully converted invasive breast cancer cells into functional adipocytes to prevent metastasis via small molecule induction of epithelial–mesenchymal transition (EMT) and re-differentiation. EMT is a well-recognized reprogramming process that can enhance cellular plasticity [95]. As previously shown, the reprogramming process to generate pluripotent cancer cells is usually associated with the potential to further differentiate into various lineages with normal cellular functions via transcription factor or small-molecule induction [95]. Thus, Ishay-Ronen et al. induced EMT by treating Py2T breast cancer cells with transforming growth factor-beta (TGF-β) in vitro and re-differentiated the reprogrammed Py2T cells into functional adipocytes by using insulin, dexamethasone, rosiglitazone, and bone morphogenetic protein 2 (BMP2). The results revealed that the reprogrammed Py2T cells could be induced to undergo adipogenesis with a cocktail of small molecules. After at least 20 days treatment with TGF-β and adipogenesis-inducing factors, the reprogrammed Py2T cells with mesenchymal characteristics expressed significantly higher C/EBPα and CCAAT/enhancer-binding protein beta (C/EBPβ), which were regulators of adipogenesis, as compared to their counterparts with epithelial characteristics. The versatile pluripotency of the reprogrammed breast cancer cells was confirmed by other mesenchymal-related differentiation including osteogenesis and chondrogenesis with detection of osteo and chondro-specific markers such as transcription factor Sp7 (Osterix), collagen type II and sex determining region Y-box 9 (SOX9). Additionally, they used MTflECad (epithelial) and MTΔCad (mesenchymal) murine models to test the efficiency and specificity of EMT-related reprogramming and re-differentiation processes. In vitro TGF-β induced reprogramming and in vivo Cre recombinase-mediated reprogramming both showed that approximately 60% of the breast cancer cells expressed C/EBPα^+^. The results confirmed that TGF-β played a vital role in regulating the EMT-related reprogramming process and mesenchymal characteristics of cancer cells both in vitro and in vivo. Moreover, combinatorial treatment with trametinib and rosiglitazone in the mouse model led to efficient adipogenesis in vivo of the reprogrammed breast cancer cells. Since trametinib and rosiglitazone are U.S. Food and Drug Administration (FDA)-approved drugs to induce EMT and adipogenesis, using them as mediators in cancer cell reprogramming therapy is clinically more feasible compared to approaches using other mediators [96]. The significant benefits of this study are not only for the identification of the small molecules in regulating breast cancer cell reprogramming and re-differentiation but also for the establishment of a replicable model which can be exploited in the evaluation of cancer cell reprogramming in many other types of cancer with different lineages. Elimination of invasive mesenchymal cancer cells by small molecule-mediated cancer cell reprogramming therapy may treat acquired chemo-/radio-resistance and cancer metastasis, but the specificity of trametinib plus rosiglitazone treatment and prevention of side effects should be further investigated in later studies [89, 96].

As noted, during cancer cell reprogramming, cellular apoptosis seems to be unaffected by the small molecules, as is cellular proliferation. For instance, the time for the G0/G1 phase is prolonged, and cell cycle-promoting genes are suppressed, indicating that the enhancement of benignity has occurred [88, 89]. Small molecule-mediated cancer cell reprogramming provides a non-viral and non-integrated approach to induce the transition from cancer cells to benign cells. As one of the potential strategies, such reprogramming approach holds great promise to effectively suppress development and relapse of various malignancies.

So far, advancements in cancer cell reprogramming are facing many challenges. First, some types of cancer (such as nasopharyngeal carcinoma) comprise of a large subpopulation of undifferentiated cancer cells, making such cancer cells difficult to be reprogrammed into benign cells due to the in situ tumor heterogeneity [97]. Direct reprogramming might be a potentially feasible approach that can be applied to these types of cancers, but there have not been any promising pieces of evidence so far [98]. Second, there are many small molecules that can serve as excellent candidates in cancer cell reprogramming in vitro, but only few of them have been officially approved by the U.S. FDA, since each small molecular drug has to be strictly reviewed based on its benefits and potential risks for the intended patients [83, 99]. Future investigations should concentrate on developing pharmacological agents-mediated cancer cell reprogramming to minimize safety and efficiency concerns. Moreover, the functions of small biochemical molecules are not sufficiently specific, suggesting potential off-target effects may sometimes happen during reprogramming [100]. Additionally, the dosage of small molecules to induce in vivo cancer cell reprogramming and differentiation should be carefully examined to avoid potentially detrimental adverse events in patients. Therefore, it is urgent to precisely decipher the molecular mechanisms of cancer cell reprogramming to alleviate the side effects. It deems to be necessary to employ an efficient delivery method for reprogramming-associated small molecules since many treatment failures have not resulted from the inefficiency of the drugs themselves, but the inefficiency of drug delivery [101].

### MicroRNA and exosome-mediated cancer cell reprogramming: emerging alternatives

It has been reported that microRNAs, including miRNA302s [102, 103], miRNA200c [103, 104], miRNA369 [103], miRNA34a [105–108], and miRNA30b [109, 110], are crucial in enhancing the expression of pluripotency-associated genes. MicroRNA has been regarded as useful biomarkers and molecular probes to target specific cell types and to manipulate cell reprogramming. However, to precisely and efficiently regulate cell transition to treat malignancies by exploiting microRNA remains challenging so far.

Lin et al. [102] first showed that human skin cancer cells could be reprogrammed into iPSCs using microRNA-302s, which are abundantly expressed in human ESCs but rapidly vanished after differentiation. It has been reported that pluripotent cancer cells with microRNA-302s transfection exhibit decreased tumorigenicity, genomic demethylation, and elevated expressions of SSEA-3/4, SOX2, NANOG, and Oct-3/4. Since the size of microRNA-302s was only approximately 1 kb, the transfection efficiency reached > 99% based on flow cytometry analyses, suggesting the size of an exogenous factor played an important role in transfection efficiency [102]. However, only 2%–5% of cancer cells were successfully reprogrammed into pluripotent ES-like cells. Gene expression analysis revealed that the pluripotent ES-like cells showed more than 86% similarity to human ES cell lines H1 and H9. Under lineage-specific differentiation-inducing media, cancer-derived ES-like cells further differentiated into benign cells, including neurons, chondrocytes, and fibroblasts. MicroRNA-200 family has also been shown to enhance EMT via targeting zinc finger E-box-binding homeobox 1 (ZEB1) axis, which is known to inhibit the tumor suppressor gene E-cadherin [104]. In human colon cancer cells, members that belong to the microRNA34 family have been proven as novel transcription targets of tumor suppressor gene p53 [105–107].

Exosomes have the capability to harbor components that mimic the constitution of the embryonic microenvironment [111]. ESC-related reprogramming factors are encased in human ESC-derived exosomes and can be delivered to cancer cells to induce the transition from malignancy to benignity. In 2017, Zhou et al. [112] demonstrated that human ESC-derived exosomes could inhibit cancer proliferation in vitro and alleviate tumorigenicity in vivo. When Colo-320 and MCF-7 cancer cell lines were cultured in ESC conditioned medium, they exhibited re-expression of pluripotency-associated markers, including Oct-4, NANOG, and SOX2 and reduction of tumorigenicity in vitro, indicating the successful reprogramming from malignancy into benignity. Approximately 90% of breast cancer cells lost Vimentin expression after exposure to ESC conditioned medium, whereas the reprogramming efficiency of the colorectal cancer cells was not determined. The results suggested that exosomes could suppress oncogenesis by promoting the expression levels of critical pluripotency-associated markers. After that, cancer cells could be reverted to a pluripotent status and restore benign differentiation pathways. However, the cancer-derived ES-like cells were not free of tumor formation in vivo, and 60% tumor size reduction was observed with cancer cells treated with ESCs-derived exosomes. Conditional medium containing exosomes inhibited cancer proliferation via prolonging the time in G1 phase, whereas lowering the time in S and G2/M phases. Zhou et al. also found that the expression level of cyclin D1 was reduced to maintain retinoblastoma hypophosphorylation after treatment of conditioned medium, leading to inhibition of G1/S phase transition [113]. Moreover, phosphorylation at serine residue 10 in the histone H3, as one of the vital epigenetic modifications during G2 phase, was significantly reduced [112]. Consistent with previous findings, various substances from human embryonic microenvironment have the potential to inhibit cancer progression and alleviate tumorigenicity in vivo [114].

## Challenges and future directions

Developments of more appropriate and efficient cancer therapy for patients remains an urgent need to more effectively combat cancer. In the era of patient-specific cancer therapy, it is being believed that reprogramming of epigenetic modifications in cancer cells has the potential to serve as a promising strategy for the global control and even ablation of cancer [13] (Table 1). Notably, cancer cell reprogramming involves the re-directing of cancer cells to generate cells with benign or less malignant functions from the in situ tumor microenvironment [59, 60, 70, 76, 88, 89, 102, 112]. The epigenetic modifications of cancer cells such as DNA methylation, and histone methylation and acetylation, are vital for cancer initiation, invasion, and recurrence. If the identification of patient-specific epigenetic states become feasible, investigators would be able to discover the weakness of cancer [82, 95].

**Table 1:** Summary of various reprogramming approaches converting cancer malignancy to benignity

Cancer type	Cell line	Reprogramming approach	Delivery method	Cell fate	Efficiency	Effects	Publication year	References
Melanoma	R545	In vitro introduction of KLF4, Oct-3/4, c-Myc	Retrovirus	Pluripotent cancer cells	0.05%–0.1%	Demethylation of the Oct-4 and NANOG promoter regions and loss of in vivo tumorigenicity in chimeras	2009	Utikal et al. [59]
Pancreatic cancer	MIAPaCa-2	In vitro introduction of KLF4, Oct-3/4, SOX2, c-Myc	Retrovirus and lentivirus	Pluripotent cancer cells	Not determined	Stable differentiation into varied lineages and loss of in vivo tumorigenicity in NOD/SCID mice	2010	Miyoshi et al. [60]
Hepatocellular carcinoma	PLC							
Colorectal carcinoma	DLD-1, HCT116							
B cell lymphoma and leukemia	RCH-ACV, CEMO-1, Val, MUTZ5, NALM-20	In vitro introduction of C/EBPα	Retrovirus and lentivirus	Macrophage-like cells	80% partially or entirely reprogrammed	Up-regulation of macrophage-associated markers and loss of in vivo tumorigenicity in immunodeficient mice	2013	Rapino et al. [70]
Hepatocellular carcinoma	HCCLM3 and Huh7	In vitro introduction of HNF1A, HNF4A, and FOXA3	Adenovirus	Hepatocyte-like cells	100% infection efficiency, reprogramming efficiency not determined	Recover of hepatocyte functions and capability of in vivo liver regeneration	2018	Cheng et al. [76]
BCR-ABL1 + precursor B-cell acute lymphoblastic leukemia	Human B-ALL clinical samples	In vitro delivery of FLT3L, IL-7, IL-3, GM-CSF, MCSF	Culture medium	Macrophage-like cells	53% initially reprogrammed; After sorting, > 98% yield	Recover of phagocytic ability and loss of in vivo tumorigenicity	2015	McClellan et al. [88]
		In vitro *i*ntroduction of C/EBPα or PU.1	Nucleofection					
Breast cancer	Py2T	In vitro delivery of TGF- β or Trametinib, in vivo EMT induction using Cre mice	Culture medium, animal model	Adipocytes	60% reprogrammed	Recover of in vitro adipocyte functions and loss of in vivo metastatic potential	2019	Ishay-Ronen et al. [89]
Skin cancer	Colo and PC3	in vitro introduction of MicroRNA-302s	Retrovirus	Pluripotent embryonic stem-like cells	100% transfection efficiency, 2%–5% reprogramming efficiency	Stable differentiation into varied lineages and loss of in vivo tumorigenicity	2008	Lin et al. [102]
Breast cancer	MCF-7 and MDA-MB-231	in vitro delivery human embryonic stem cells-derived exosomes	Culture medium	Pluripotent embryonic stem-like cells	90% of breast cancer cells lost Vimentin expression	Recover of benign differentiation pathways and reduction of in vivo tumor-forming potential	2017	Zhou et al. [112]
Colorectal carcinoma	Colo-320 and HT-29							

Since the invention of iPSC technology by Yamanaka et al. in the year 2006, many cell reprogramming approaches that are capable of regulating cell fate decisions have been proposed. Based on decades of basic and clinical researches concentrating on deciphering the epigenetic passcode of cancer, clinical translation of cancer cell reprogramming has been quickly fueled. Nevertheless, it also comes to an agreement that recent investigations have only scraped a superficial layer of cancer epigenetics [13, 19]. One of the most frequently occurred epigenetic lesions is genome-scale loss of DNA methylation at gene promoters of oncogenes and hypermethylation of tumor suppressor genes, leading to the up- and down-regulation of those genes in a transcriptional level so that cancer cells can escape from growth and survival control checkpoints [19]. To effectively manipulate such epigenetic lesions to induce cancer cell reprogramming, it is necessary to continuously obtain a comprehensive understanding of the reprogramming mechanisms from a molecular perspective. There exists a large subpopulation of epigenetically blocked tumor suppressor genes in cancer cells [13, 20, 82]. If cancer cell reprogramming could safely, specifically and effectively re-activate those silenced tumor suppressor genes, current cancer treatment could be significantly advanced.

## Conclusion

Currently, it is only feasible to conduct such reprogramming in laboratory settings, but there is an increasing number of studies that are focusing on optimizing cancer cell reprogramming so that it can be safely, specifically and effectively used in clinical treatments [96]. Various approaches, including transcription factor-, small molecule-, microRNA-, and exosome-mediated cancer cell reprogramming, have achieved tremendous accomplishments and ingenuity, making it increasing versatile and convenient in pre-clinical as well as clinical practices. Although genetic mutations have been deemed as a cancer-initiating event, it remains technically and ethically challenging to apply gene therapy in humans. Epigenetic alterations in cancer are also involved in cancer initiation and progression, but unlike genetic mutations, the epigenetic states of cancer can be effectively reprogrammed via distinct approaches.

Ultimately, safety, specificity, and efficiency trials in murine models and other animal models will be entailed in the future to confirm the in vivo therapeutic potential for cancer cell reprogramming. Challenges for cancer cell reprogramming not only involve the in vivo dosage and delivery but also the instability of reprogrammed cancer cells and potential off-target effects. In summary, challenges ahead in cancer cell reprogramming is currently impeding the progress to translate the potentially promising approach to clinical applications, but they appear to be solvable based on rapidly evolving frontier in cancer biology.

## Data Availability

Not applicable.

## Abbreviations

CSC: cancer stem cell
ESC: embryonic stem cell
iPSC: induced pluripotent stem cell
KLF4: Kruppel-like factor 4
Oct-3/4: Octamer-binding transcription factor 3/4
SOX2: sex-determining region Y-box 2
SSEA-1: stage-specific embryonic antigen-1
TRA-1–60: T cell receptor alpha-1–60
TRA-1–81: T cell receptor alpha-1–81
ROCK: Rho-associated protein kinase
VPA: valproic acid
RepSOX2: 2-[3-(6-methyl-2-pyridinyl)-1*H*-pyrazol-4-yl]-1,5-naphthyridine
OAC1: Oct4-activating compound 1
MET: mesenchymal–epithelial transition
5-Fu: 5-fluorouracil
LIN28: Lin-28 homolog
BCR: breakpoint cluster region
ABL1: Abelson murine leukemia viral oncogene homolog 1
B-ALL: B cell acute lymphoblastic leukemia
C/EBPα: CCAAT/enhancer-binding protein alpha
HCC: hepatocellular carcinoma
HNF1A: hepatocyte nuclear factor 1 alpha
HNF3A: hepatocyte nuclear factor 3 alpha
FOXA3: forkhead box protein A3
ALB: albumin
AFP: alpha-fetoprotein
EpCAM: epithelial cell adhesion molecule
FLT3L: FMS-like tyrosine kinase ligand
IL-7: interleukin 7
IL-3: interleukin 3
GM-CSF: granulocyte-macrophage colony stimulating factor
MCSF: macrophage colony-stimulating factor
EMT: epithelial–mesenchymal transition
TGF-β: transforming growth factor beta
BMP2: bone morphogenetic protein 2
C/EBPβ: CCAAT/enhancer-binding protein beta
SOX9: sex determining region Y-box 9
FDA: Food and Drug Administration
ZEB1: zinc finger E-box-binding homeobox 1
Osterix: transcription factor Sp7
